# Chylopericardium due to Subclavian Vein Thrombosis in the Setting of Protein S Deficiency

**DOI:** 10.1155/2021/2232057

**Published:** 2021-11-28

**Authors:** Ian Jackson, Yaman Alali, Abedel Rahman Anani, Ali Nayfeh, Arindam Sharma, Abhishek Thandra, Amjad Kabach

**Affiliations:** ^1^Department of Medicine, Creighton University School of Medicine, Omaha, NE, USA; ^2^Department of Pulmonary and Critical Care, Creighton University School of Medicine, Omaha, NE, USA; ^3^Department of Cardiology, Creighton University School of Medicine, Omaha, NE, USA

## Abstract

**Background:**

Chylopericardium is the accumulation of lymphatic fluid in the pericardial cavity. It can be idiopathic or secondary to trauma, cardiothoracic surgery, neoplasm, radiation, tuberculosis, lymphatic duct dysfunction, thrombosis, or other causes. We present a case of chylopericardium due to subclavian vein thrombosis in a patient with protein S deficiency. *Clinical Case*. A 48-year-old man with a history of protein S deficiency presented to the emergency department with shortness of breath and a productive cough. CT of the chest showed pulmonary emboli, moderate pericardial effusion, and a large thrombus of the superior vena cava, brachiocephalic vein, and subclavian veins. He developed echocardiographic evidence of cardiac tamponade so he underwent pericardiocentesis with drainage of milky-appearing fluid. Analysis of the fluid showed elevated triglycerides consistent with chylopericardium. The pericardial effusion reaccumulated, likely secondary to lymphatic duct obstruction due to his subclavian vein thrombus. Catheter-assisted thrombolysis was performed with resolution of the patient's effusion and symptoms.

**Conclusion:**

Chylopericardium is a rare but important complication of subclavian vein thrombosis. Management is typically with surgical intervention, although our case represents successful treatment with catheter-assisted thrombolysis.

## 1. Introduction

Chylopericardium is a rare condition characterized by the accumulation of milky fluid rich in triglycerides and chylomicrons in the pericardial cavity. Chylopericardium can be idiopathic or secondary to injury or obstruction of the thoracic duct. The most common causes of secondary chylopericardium are trauma and cardiac or thoracic surgery. Less common causes include neoplasm, radiation therapy, tuberculosis, lymphangiectasis, lymphangioma, cystic hygroma, filariasis, and subclavian vein thrombosis [1, 2].

## 2. Case Description

A 48-year-old man presented to the emergency department with shortness of breath and a productive cough that had been gradually worsening for the past one week. He had a history of protein S deficiency and numerous associated deep vein thromboses and pulmonary emboli requiring placement of an inferior vena cava filter and long-term anticoagulation. On arrival to the emergency department, he had a heart rate of 101, respiratory rate of 31, blood pressure of 153/110 mmHg, and oxygen saturation of 89% on room air. Physical examination showed lower extremity edema and labored breathing.

**Figure 1:**
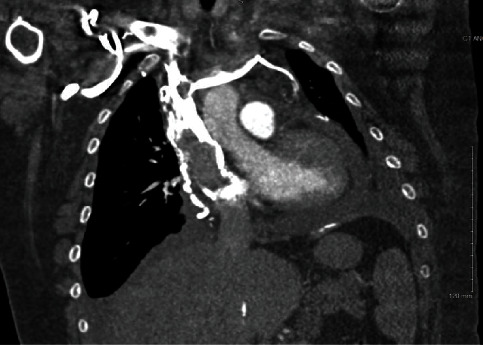
CT of the chest with SVC and subclavian thrombus.

**Figure 2:**
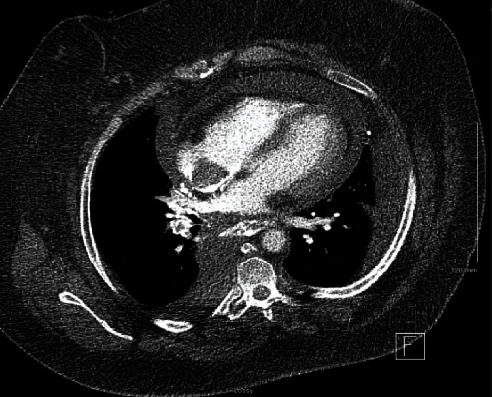
CT of the chest with right atrial thrombus and pericardial effusion.

Laboratory workup was significant for creatinine of 1.47 mg/dL (normal range 0.60–1.30 mg/dL). A respiratory pathogen screen, severe acute respiratory syndrome coronavirus (COVID-19) PCR testing, and troponin were negative. An electrocardiogram (EKG) showed sinus tachycardia without evidence of ischemia. Computed tomography (CT) of the chest showed pulmonary emboli with involvement of the right middle and lower lobar arteries, a moderate pericardial effusion, and a large, nearly occlusive thrombus in the superior vena cava (SVC) with extension into the right atrium and bilateral brachiocephalic veins. Extensive venous collateralization was also evident and thought to be indicative of obstruction in venous return (Figures 1 and 2). The patient was treated with supplemental oxygen and a heparin infusion.

**Figure 3:**
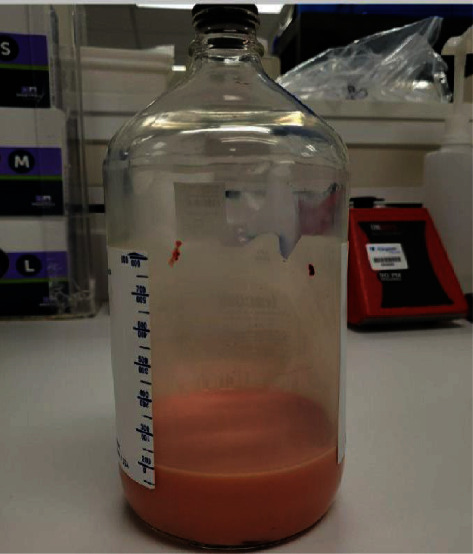
Chylous pericardial fluid.

Vascular surgery determined that the patient's SVC thrombus was likely acute on chronic as it was present but not noted on previous imaging. An initial echocardiogram showed a moderate effusion without evidence of tamponade. However, serial echocardiograms demonstrated increasing size of the effusion with development of right ventricular dilation and collapse. Pericardiocentesis was performed with drainage of 400 mL of chylous-appearing fluid (Figure 3).

Analysis of the pericardial fluid showed 91,000 red blood cells/*μ*L; 26,248 nucleated cells/*μ*L with 62% neutrophils, 36% lymphocytes, and 2% monocytes; protein of 4.8 gm/dL; albumin 2.1 gm/dL; lactate dehydrogenase 373 U/L; glucose 158 mg/dL; amylase 20 U/L; lipase 25 U/L; cholesterol < 200 mg/dL; and triglycerides of 2,726 mg/dL. Gram stain and culture of the fluid were negative for organisms. Cytology was negative for malignancy. Given the results of the pericardial fluid analysis, it was determined that the chylous pericardial effusion was likely due to lymphatic obstruction secondary to subclavian vein thrombus.

The patient was treated with therapeutic doses of enoxaparin for anticoagulation during hospital admission. After pericardiocentesis, he was monitored with serial echocardiograms which showed gradual reaccumulation of his pericardial effusion without evidence of tamponade. A repeat CT of the chest showed worsened venous obstruction with complete occlusion of the left subclavian vein. Vascular and cardiothoracic surgery teams decided that the best treatment option was definitive management of the patient's thrombus as a pericardial window would result in continuous effusion. Despite a high risk of causing pulmonary embolism, the patient underwent catheter-assisted thrombolysis directed at the left subclavian thrombosis.

Following the procedure, a repeat CT angiogram of the chest showed a decrease in clot burden. The patient clinically improved and was discharged without further operative intervention. He was found to have only trace pericardial effusion on echocardiography with no residual symptoms at one-year follow-up.

## 3. Discussion

Secondary chylopericardium is usually due to malignancy or trauma to the thoracic duct during surgery [3]. Rarely, it has been reported to occur secondary to thrombosis of the superior vena cava and subclavian veins, typically due to catheter-related thrombosis [1–3]. One case reported chylopericardium secondary to superior vena cava and brachiocephalic vein thrombosis following community-acquired pneumonia [4]. However, no previous case reports describe secondary chylopericardium due to a hereditary thrombophilia. We report a very rare case of chylopericardium secondary to SVC and left subclavian vein thrombosis in a patient with protein S deficiency.

The cardiac lymphatic vessels normally join as a common trunk and drain into the thoracic duct. In the presented case, venous thrombosis of the SVC, bilateral brachiocephalic veins, and the left subclavian vein likely led to occlusion of ostium of the thoracic duct and consequent increase in thoracic duct pressure [5]. This may have caused reflux of chylous lymph through the lymphatics draining the heart and pericardium, resulting in chylopericardium. This mechanism has been described previously in the literature. Normally, the lymphatic vessels have valves that preclude retrograde flow of chyle unless the pressure increases to the range of 35 to 50 cm water [6, 7]. Additionally, experiments have shown that blocking the thoracic duct alone does not cause chylopericardium due to the presence of several collateral pathways within the venous system, such as the azygous vein and superior vena cava. This indicates the importance of mechanical venous obstruction in the pathophysiology of chyloperdicardium [6, 7]. This also may explain why the presence of collateral venous circulation evident on our patient's chest CT may have prevented the reaccumulation of chyle in the pericardium.

Chylopericardium presents similarly to other causes of pericardial effusion. It varies considerably, ranging from asymptomatic to cardiac tamponade. Fatigue, dyspnea, and cough are the most common symptoms [1–3]. Initial workup includes a chest X-ray which may show an enlarged cardiac silhouette and echocardiography which can confirm the presence of pericardial effusion and evaluate for cardiac tamponade. Pericardiocentesis can be used for both diagnostic and therapeutic purposes. Analysis of the pericardial fluid in chylopericardium typically shows high levels of triglycerides and protein with an elevated lymphocyte count.

Further investigations to determine the underlying cause of chylopericardium should also be pursued. A thorough history can evaluate for recent trauma, thoracic surgery, potential exposure to tuberculosis, or signs of malignancy [1, 2]. Testing for tuberculosis was not done in the presented case as there was no clinical or radiographical evidence to suggest tuberculosis and the patient had no history of travel outside the United States. A lymphangiogram may also be useful in the diagnosis of mediastinal lymphangiomatosis; however, this was not done in this case as the underlying cause for his chylopericardium was established.

A scoring system has been suggested by Dib et al. to assist in the diagnosis of chyloperdicardium [3]. The score is calculated by assigning a point to each of the following features: milky yellowish appearance of fluid, triglyceride level of >500 mg/dL, total cholesterol to triglyceride ratio of <1, and negative fluid bacterial culture with lymphocytic predominance on fluid cell count. A score of two is required for a diagnosis of chylopericardium. The patient in the presented case had a score of four, which is highly indicative of chylopericardium.

Initial management of chylopericardium includes a low-fat diet and initiation of medium-chain triglycerides, which are absorbed via the portal vein rather than via the lymphatic vessels [1–3]. Pericardiocentesis is both diagnostic and therapeutic. Surgical intervention, including pericardial window or thoracic duct ligation, is generally recommended when the daily pericardial drainage is more than 1,500 mL daily, drainage is more than 500 mL daily for five days, or in the setting of malnutrition or recurrent chylopericardium [1, 7].

In the presented case, the cause of chylopericardium was attributed to the extensive venous thrombosis of the patient's SVC and left subclavian vein. Despite anticoagulation with therapeutic enoxaparin, serial imaging showed increasing size of pericardial effusion with evidence of tamponade and progressive thrombosis. Similar cases in the literature were primarily treated with venous stenting or surgical intervention [4, 8]. Although surgical intervention was considered in this case, it was felt that this would only prevent reaccumulation of chyle without addressing the underlying cause. Treatment with catheter-directed thrombolysis was instead pursued. The patient's symptoms resolved, and his chylopericardium did not recur, indicating successful treatment.

## 4. Conclusion

Chylopericardium is a rare complication of SVC or subclavian vein thrombosis. Our patient with protein S deficiency represents a very rare case of chylopericardium caused by thrombosis secondary to hereditary thrombophilia. Our case also demonstrates that systemic anticoagulation and catheter-directed thrombolysis may be sufficient for treatment without the need for surgical intervention.

## Data Availability

No additional data was accessed or generated for this manuscript.
